# Bioequivalence of two tablet formulations of cefpodoxime proxetil in beagle dogs

**DOI:** 10.3389/fvets.2022.1048823

**Published:** 2022-10-14

**Authors:** Yan-Yan Gao, Ka-Na Sang, Peng-Peng Li, Jie Hao, Cong Zhang, Huan-Juan Li, De-Gang Zhou

**Affiliations:** ^1^National Research Center for Veterinary Medicine, Luoyang, China; ^2^Luoyang Huizhong Animal Medicine Co., Ltd., Luoyang, China

**Keywords:** pharmacokinetics, bioequivalence, tablets, cefpodoxime proxetil, dogs

## Abstract

The pharmacokinetic profiles and bioequivalence of two cefpodoxime proxetil tablets were investigated in Beagle dogs. A single-dose, four-way complete replication and crossover design was used in the present study. A total of 28 healthy Beagle dogs (half male and female) with an average body weight of 11.1 kg were randomly allocated to this study. A whole reference or test tablet containing the equivalent of 100 mg of cefpodoxime was administered orally to each dog. Serial plasma samples were collected, and cefpodoxime concentrations were determined by ultra-performance liquid chromatography-mass spectrometry (UPLC-MS/MS). Then a non-compartmental method was used to calculate the pharmacokinetic parameters of both tablet formulations. The average bioequivalence (ABE) or reference-scaled average bioequivalence (RSABE) methods were used to determine the 90% confidence interval (CI) of AUC_INF_obs_ and C_max_. No significant differences were observed for both parameters between both tablets. The test formulation was bioequivalent to the reference one because the 90% CI ranges of C_max_ and AUC_INF_obs_ were all between 80 and 125%.

## Introduction

In veterinary clinics, cefpodoxime proxetil is the most often prescribed oral third-generation cephalosporin antibiotic (1). It is a prodrug ester created expressly to be stable in the stomach, and intestinal brush border enzymes would convert it to the active cefpodoxime (2). Cefpodoxime exhibits good activity against many gram-positive and gram-negative organisms and good tissue penetration. It prevents the synthesis of bacterial cell walls, just like other cephalosporins. Its covalent attachment to penicillin-binding proteins (PBPs), which are necessary for the synthesis of bacterial cell walls, is the primary cause of this interference. Cefpodoxime proxetil is therefore regarded as antibacterial. It was also stable toward the most commonly found plasmid-mediated beta-lactamases (3).

Dogs with skin infections (wounds and abscesses) caused by various bacteria, including *Staphylococcus intermedius, Staphylococcus aureus, Streptococcus canis, E. coli, Proteus mirabilis*, and *Pasteurella multocida*, are frequently treated with cefpodoxime proxetil (4–7). The minimum inhibitory concentration (MIC) values of cefpodoxime varied from 0.06 to 256 μg/mL against *E. coli* isolates (*n* = 2,392), with MIC_50_ and MIC_90_ values of 0.5 and 32 μg/mL, respectively (8). Cefpodoxime had a lower rate of resistance (13 vs. 98% for cephalothin, 48% vs. ampicillin, 40% vs. amoxicillin-clavulanic acid, and 18% vs. ticarcillin-clavulanic acid) than other cephalosporins (8). Similar MIC values, ranging from 0.12 to 256 μg/mL, were also reported for cefpodoxime against canine *E. coli* isolates (*n* = 301) (9). However, higher MIC values were found for cefpodoxime proxetil, which were 53.33, 85.33, 53.33, 26.67, 42.67, 85.33, and 42.67 μg/mL against *E. coli, Salmonella, Proteus mirabilis, Pasteurella, Shigella dysenteriae, Staphylococcus*, and *Streptococcus agalactiae*, respectively (10). It should be noted that the isolated source of those strains was not available. And we cannot confirm whether the bacteria were isolated from dogs or other animals.

The tablet form of cefpodoxime proxetil for dogs was authorized by the US Food and Drug Administration (US FDA) in July 2004 under the brand name SIMPLICEF^®^ (11). A similar cefpodoxime proxetil tablet indicated for use in adult and pediatric human patients was Vantin^®^ (12). The plasma pharmacokinetics of oral cefpodoxime proxetil and intravenous cefpodoxime sodium have been examined in beagle dogs to employ cefpodoxime in canine clinics intelligently (12). Additionally, the pharmacokinetics in plasma and subcutaneous fluid were studied after giving cefpodoxime proxetil to beagle dogs orally (4). But only Vantin^®^ was investigated in those studies. Both Vantin^®^ and SIMPLICEF^®^ are expensive and are unavailable in the Chinese market, which limits the treatment of skin infections and jeopardizes the welfare of dogs in China. In order to expand the range of treatments available in China for canine skin infections, a cefpodoxime proxetil tablet has been produced by Luoyang Huizhong Animal Medicine Co., Ltd. (Luoyang, China). Therefore, the current study aimed to compare the pharmacokinetic profiles of domestically produced cefpodoxime proxetil with SIMPLICEF^®^ (Zoetis) to assess their bioequivalence and, ultimately, the potential for substitution in dogs.

## Materials and methods

### Chemical reagents

The test product was the cefpodoxime proxetil tablet (Lot No. ZS20191101) manufactured by Luoyang Huizhong Animal Medicine Co., Ltd. (Luoyang, China). And SIMPLICEF^®^, purchased from Zoetis (Lot No. JH8427), served as the reference product. Both test and reference tablets contain cefpodoxime proxetil equivalent to 100 mg cefpodoxime. The analytical standard for cefpodoxime (Lot No. 10-KMT-108-3) with a purity of 95% (calculated as cefpodoxime moiety) was purchased from Toronto Research Chemicals Inc (ON, Canada). Acetonitrile, methanol, and formic acid were HPLC grade and supplied by Tianjin Kemi O Chemical Reagent Co., Ltd. (Tianjin, China). Deionized water was purified using a Milli-Q system (Millipore, Milford, MA, USA).

### Animals

A total of 28 healthy Beagle dogs (half male and female) provided by Beijing Amersey Biotechnology Co., Ltd. (Beijing, China) were enrolled in the present study. Their body weights ranged from 10.1 to 12.05 kg. All dogs were subjected to physical examinations by a veterinary to ensure their health. Animals were housed individually in large wire cages (130 × 100 × 120 cm) in a room where temperature and humidity could be controlled. The room temperature was maintained between 24 and 28°C with relative humidity at 40–50%. All dogs were fed a dog food for all life stages (Pholiton^®^) twice daily and given water ad libitum. These dogs were acclimated to experimental conditions for 1 week, during which neither vaccines nor drugs were given. Animal experiments were conducted under protocols approved by the local Institutional Animal Care and Use Committee (IACUC), and the approved # was 13303-19-E-004.

### Study design

Because cefpodoxime proxetil tablet has been proved as a highly variable veterinary drug (13), this study was performed in a single-dose, four-way complete replication and crossover design (14, 15). All dogs were randomly and equally divided into two groups [Reference-Test-Reference-Test (RTRT) and Test-Reference-Test-Reference (TRTR) groups]. Reference and test tablets were alternately given to both groups in four phases. Specifically, in phases 1 and 3, dogs in the RTRT group orally received reference tablets, while those in the TRTR group were orally administered test tablets; in phases 2 and 4, both groups received different cefpodoxime proxetil tablets in phases 1 and 3. All dogs were fasted for 16 h before and 8 h after drug administration. Between each phase, a washout period (1 week) was scheduled. A whole tablet containing the equivalent of 100 mg of cefpodoxime was administered to each dog at each dose. The dose was ~9 mg/kg of body weight because the average body weight of dogs was 11.1 kg.

Blood samples (~2 mL) were collected into heparinized tubes *via* the cephalic brachial vein at 0 (before administration), 0.5, 1, 1.5, 2, 2.5, 3, 5, 8, 12, 24, and 36 h after dosing. The plasma was obtained by centrifugation at 3,000 g for 10 min and stored at −20°C until analysis.

### Analytical method

The recommended guidance by US FDA was used to develop and validated the current analytical method (16). Cefpodoxime concentrations were determined using a UPLC-MS/MS analytic method. Briefly, 100 μl of plasma was mixed with 10 μl of acetonitrile, followed by vortexing for 10 s, then added 300 μl of acetonitrile: methanol (1:1), vortexed for 2 min, and centrifuged at 12,000 g for 20 min at 4°C. The supernatant (3.5 μl) was subjected to UPLC-MS/MS analysis (Waters Acquity UPLC and Water Quattro Premier; Waters Co, USA). The chromatographic separation was performed with a reverse-phase C18 analytical column (50 × 2.1 mm inner diameter; Kinetex 2.6 μm C18 100 Å; Phenomenex Inc.) maintained at 25°C. The mobile phase consisted of two solutions, including solvent A (0.1% formic acid in water) and solvent B (0.1% formic acid in methanol), with a flow rate of 0.35 mL/min. The UPLC gradient elution profile was applied as follows: 0–0.3 min, isocratic 98% A; 0.3–0.9 min, 98% A to 2% A; 0.9–1.5 min, isocratic 2% A; 1.5–1.51 min, 2% A to 98% A; 1.51–3 min, isocratic 98% A.

For mass spectrometry analysis, data was acquired using electrospray ionization (ESI) in the positive mode; the capillary voltage was set at 3,500 V. The ion source and desolvation temperatures were maintained at 120 and 350°C, respectively. The mass spectrometer was operated in MS/MS mode using multiple reactions monitoring (MRM) mode.

A stock solution of cefpodoxime was prepared in dimethyl sulfoxide at a concentration of 1 mg/mL. A series of working solutions with a concentration range of 200–50,000 ng/mL was obtained by diluting the stock solution with acetonitrile. Calibration curve samples were prepared by the matrix-matched standard calibration method, with concentrations ranging from 20 to 5,000 ng/mL. The calibration standard curve was Y = 35.8736 X−105.917 (*R*^2^ = 0.9974), where X and Y were cefpodoxime concentration and chromatographic peak area, respectively. Samples with concentrations above the up limit of the concentration range were first diluted using the mobile phase, then quantified as before. For precision and accuracy, six replicates at four different concentrations (20, 50, 500, and 4,000 ng/mL) were tested to evaluate coefficients of variation (CV) and recoveries, respectively. And the average extraction recovery of cefpodoxime in plasma was 103.74% (Supplementary Table 1), and the intra- and inter-day variation coefficients ranged from 4.51 to 10.70% and from 7.65 to 9.49%, respectively (Supplementary Table 2). The limits of quantification (LOQ) and detection (LOD) were determined based on signal-to-noise ratios of ≥10 and ≥3, whose values were 20 and 10 ng/mL, respectively. We also tested the stability of cefpodoxime. Cefpodoxime was spiked in plasma at three different concentrations (50, 400, and 4,000 ng/mL), and these spiked plasma samples were freeze-thawed three times or stored at room temperature for 24 h. We compared the effect of a different number of freeze-thaw treatments (Supplementary Table 3) and room temperature storage (Supplementary Table 4) on the stability of cefpodoxime.

### Data analysis

The 90% confidence interval (CI) is advocated by the Center for Veterinary Medicine (CVM) in the US FDA as the best available method for evaluating bioequivalence data. And the critical parameters for bioequivalence are AUC_INF_obs_ and C_max_ (17). A non-compartmental analysis method (18) was used to determine these pharmacokinetic parameters using Phoenix WinNonLin software (Version 8.1; Certara) (19), then the average bioequivalence (ABE) analysis was performed based on the guidance by US FDA (17). Mixed model analysis was used to estimate upper and lower bounds for all three parameters of both formulations. The recommended bioequivalent limit is 80–125% (17, 20). For a pharmacokinetic parameter, if its within-subject standard deviation (S_WR_) is above 0.294, its within-subject coefficient of variation (CV_W_%) will be >30%. In this case, the reference-scaled average bioequivalence (RSABE) method was used to perform the bioequivalence analysis (21).

## Results

**Figure 1:**
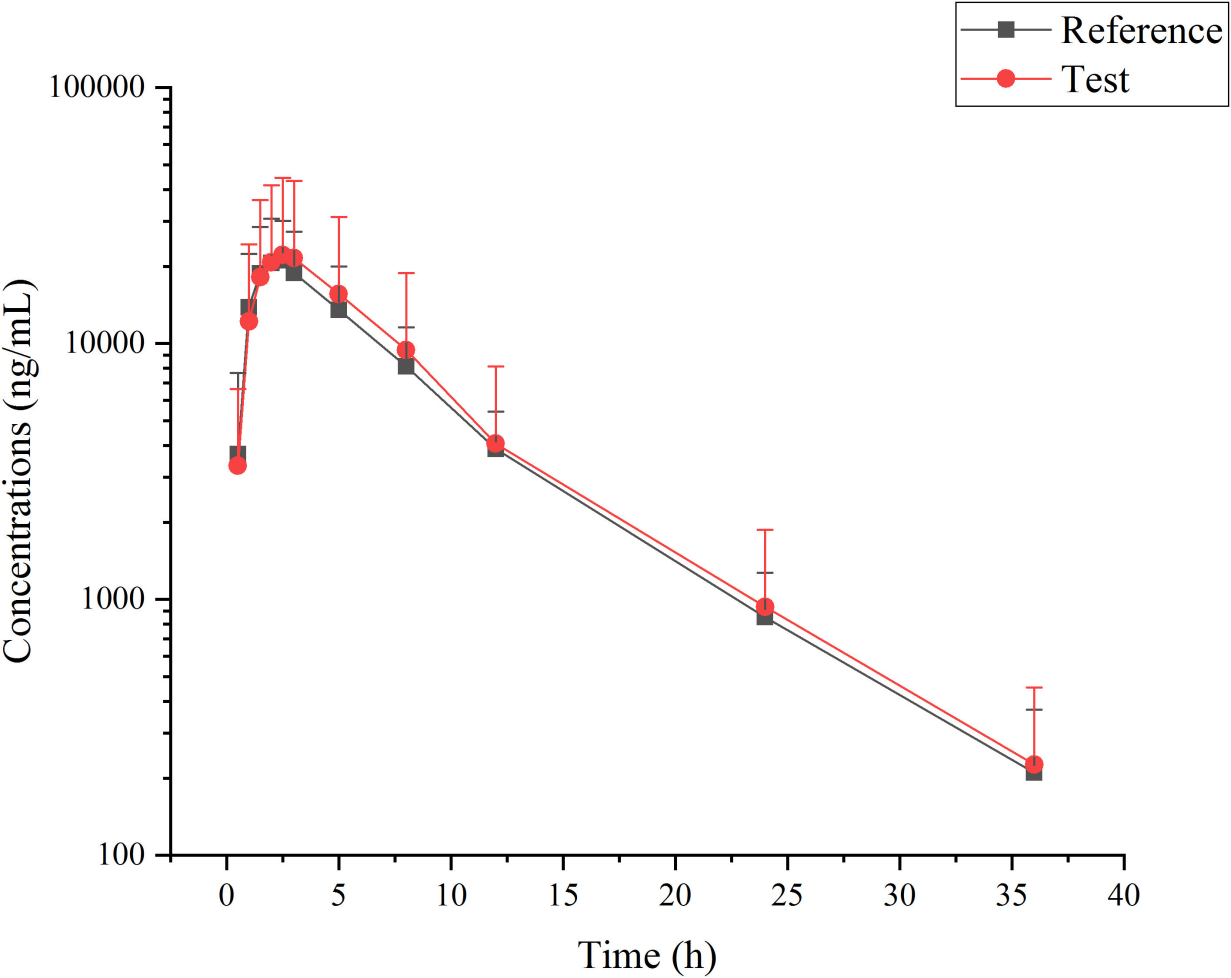
Cefpodoxime concentrations in Beagle dogs following single administration of reference and test tablet formulations of cefpodoxime proxetil.

Cefpodoxime was detectable within 36 h following a single oral administration of an intact cefpodoxime proxetil tablet. The average plasma concentration-time curves corresponding to both the test and the reference products are presented in Figure 1. The pharmacokinetic parameters were calculated using a non-compartmental method and are shown in Table 1. The S_WR_ values were calculated as 0.381 and 0.281 for ln (C_max_), and ln (AUC_INF_obs_), respectively. Therefore, the ABE method was used to perform the bioequivalent analysis for AUC_INF_obs_, while the RSABE method was used for C_max_. All results of the bioequivalence analysis are shown in Table 2, which indicates that the test and reference formulations are bioequivalent.

**Table 1:** Pharmacokinetic parameters calculated for two tablet formulations of cefpodoxime proxetil in beagle dogs.

**Parameters**	**Unit**	**Reference**	**Test**
		**formulation**	**formulation**
C_max_	ng/mL	23,451 ± 9,585	24,868 ± 9,475
AUC_INF_obs_	h·ng/mL	169,439 ± 50,980	184,649 ± 56,209

C_max_, observed peak concentration; AUC_INF_obs_, area under the concentration-time curve from 0 to infinity.

**Table 2:** Bioequivalent analysis of both tablet formulations of cefpodoxime proxetil in beagle dogs.

**Parameters**	**Ratio_%Ref_**	**90% CI range**	
		**Lower**	**Upper**
		**limit (%)**	**limit (%)**
ln (C_max_)	107.55	95.86	120.68
ln (AUC_INF_obs_)	109.77	99.26	121.40

The parameter of ln (C_max_) was analyzed using the RSABE method, while ln (AUC_INF_obs_) was done through an ABE method.

## Discussion

No discernible variations were seen for any pharmacokinetic parameters following oral administration of the cefpodoxime proxetil reference and test tablet formulations (Table 1). The test formulation was bioequivalent to the reference formulation, as shown by the 90% CI ranges of C_max_ and AUC_INF_obs_, which were both between 80 and 125% (Table 2).

The present C_max_ values (23,451 and 24,868 ng/mL, respectively), for both reference and test formulation were higher than those previously reported in dogs, which were 17.8 and 16.4 μg/mL for suspension and tablet formulations of cefpodoxime proxetil, respectively (12). However, higher C_max_ values (27.14 ± 4.56 and 32.96 ± 6.92 μg/mL) were seen in beagle dogs orally given the same dosage (4, 22). These variations in C_max_ may result from various sampling strategies and detection techniques. The current T_max_ was found to be 2.55 ± 1.26 and 2.54 ± 1.33 h after dosing a whole reference and test tablet, which were both later than those previously reported for suspension and tablet (2 and 2.21 h, respectively) (12). However, a later T_max_ (3 ± 1.1) was previously recorded for cefpodoxime after oral administration of a mean dose of 9.6 mg/kg (22). And a later T_max_ (3.5 h) was demonstrated at a lower oral dose (5 mg/kg) in dogs (4). Different T_max_ and C_max_ have been recorded in various species, with rabbits reporting values of 0.91–0.93 μg/mL and 2.7–2.9 h, respectively (23), and rats reporting values of 5.886–19.771 μg/mL and 2.302–3.480 h, respectively (24).

The apparent elimination half-life (HL_Lambda_z) for the reference and test formulations, respectively, was determined to be 5.67 ± 2.32 and 5.64 ± 2.46 h, which was consistent with previously published values ranging from 5.61 (12) to 5.75 h (22). The current values were, however, longer than those of another cefpodoxime proxetil tablet (Vantin^®^), ranging from 3.01 to 4.72 h (4). Shorter HL_Lambda_z values have also been recorded in rabbits [2.72–2.83 h; (23)] and rats [1.741–5.318 h; (24)]. The estimated AUC_INF_obs_ values for the reference and test formulations were 169.439 and 184.649 h·μg/mL, respectively (Table 1), which were greater than those (107 and 145 h·μg/mL) previously reported in dogs (4, 12).

The current study included a four-way, single-dose, crossover design. However, a prior bioequivalence study in rabbits utilized a two-way, single-dose, and crossover design (23). Cefpodoxime has been demonstrated to be a highly variable drug (11), which is why a four-way method was used in this investigation (21). The current S_WR_ values for ln (C_max_) and ln (AUC_INF_obs_) were calculated to be 0.381 and 0.281, respectively, indicating large variability. In dogs, there have been reports of greater fluctuations in the pharmacokinetic properties of cefpodoxime tablets. The average peak concentration and its SD were calculated to be 16.4 and 11.8 μg/mL, respectively (12).

## Conclusion

It has been established that cefpodoxime has quick absorption and slow excretion. The relative bioavailability of the test formulation was calculated to be 108.9%. Additionally, the test formulation was bioequivalent to the reference formulation.

## Data availability statement

The original contributions presented in the study are included in the article/Supplementary material, further inquiries can be directed to the corresponding author/s.

## Ethics statement

The animal study was reviewed and approved by the Local Institutional Animal Care and Use Committee (IACUC).

## Author contributions

D-GZ conceived this project. Y-YG, K-NS, and P-PL performed the pharmacokinetics experiments. JH and CZ determined the cefpodoxime concentrations in collected samples. Y-YG, H-JL, and D-GZ performed the bioequivalent analysis. Y-YG wrote this manuscript. All authors read and approved this final manuscript.

## Conflict of interest

Authors JH, CZ, H-JL, and D-GZ were employed by Luoyang Huizhong Animal Medicine Co., Ltd. The remaining authors declare that the research was conducted in the absence of any commercial or financial relationships that could be construed as a potential conflict of interest.

## Publisher's note

All claims expressed in this article are solely those of the authors and do not necessarily represent those of their affiliated organizations, or those of the publisher, the editors and the reviewers. Any product that may be evaluated in this article, or claim that may be made by its manufacturer, is not guaranteed or endorsed by the publisher.
