# Supplementary material for: Bioequivalence of two tablet formulations of cefpodoxime proxetil in beagle dogs
Source: Front Vet Sci. 2022 Oct 14;9:1048823. doi: 10.3389/fvets.2022.1048823 (PMC9614231; doi:10.3389/fvets.2022.1048823)
Supplement: Supplementary file 1 [file Data_Sheet_1.docx]

**Bioequivalence of Two Tablet Formulations of Cefpodoxime Proxetil in Beagle Dogs**

Yan-Yan Gao^1^, Ka-Na Sang^1^, Peng-Peng Li^1^, Jie Hao^2^, Cong Zhang^2^, Huan-Juan Li^2^, De-Gang Zhou^1,2,*^

1. National Research Center for Veterinary Medicine, Luoyang, 471023, China

2. Luoyang Huizhong Animal Medicine Co., Ltd., Luoyang, 471023, China

**Supplementary Tables**

**Table S1.** Recovery of cefpodoxime spiked at different concentrations in plasma

| Concentrations (ng/mL) | Day1 | Day2 | Day3 |
| --- | --- | --- | --- |
| 20 | 97.77 | 102.21 | 104.37 |
|  | 95.8 | 110.35 | 106.58 |
|  | 107.17 | 90.27 | 118.97 |
|  | 91.94 | 112.85 | 109.58 |
|  | 83.37 | 95.52 | 90.53 |
|  | 90.17 | 104.04 | 107.81 |
| 50 | 102.36 | 102.04 | 97.94 |
|  | 116.65 | 103.12 | 99.94 |
|  | 115.9 | 97.69 | 99.7 |
|  | 114.18 | 103.2 | 110.78 |
|  | 113.34 | 97.85 | 90.11 |
|  | 116.46 | 91.26 | 109.74 |
| 500 | 115.7 | 102.36 | 103.78 |
|  | 114.54 | 110.72 | 108.09 |
|  | 113.74 | 97.58 | 91.87 |
|  | 108.88 | 111.21 | 110.89 |
|  | 88.49 | 107.31 | 114.38 |
|  | 97.93 | 115.59 | 108.12 |
| 4000 | 114.88 | 113.78 | 106.07 |
|  | 90.72 | 105.06 | 107.61 |
|  | 93.56 | 113.22 | 109.21 |
|  | 98.16 | 109.37 | 105.31 |
|  | 101.8 | 97.68 | 95.94 |
|  | 84.84 | 92.16 | 107.27 |

**Table S2. Recovery and coefficient of variation of cefpodoxime spiked in plasma**

| Concentrations (ng/ml) | Day | Recovery (%) | Intra-day CV (%)（n=6） | Inter-day CV (%)（n=6） |
| --- | --- | --- | --- | --- |
| 20 | 1 | 94.37 | 8.50 |  |
|  | 2 | 102.54 | 8.38 | 9.49 |
|  | 3 | 106.31 | 8.68 |  |
| 50 | 1 | 113.15 | 4.81 |  |
|  | 2 | 99.19 | 4.65 | 8.15 |
|  | 3 | 101.37 | 7.67 |  |
| 500 | 1 | 106.55 | 10.33 |  |
|  | 2 | 107.46 | 6.09 | 7.65 |
|  | 3 | 106.19 | 7.38 |  |
| 4000 | 1 | 97.33 | 10.70 |  |
|  | 2 | 105.21 | 8.31 | 8.48 |
|  | 3 | 105.24 | 4.51 |  |

**Table S3. Stability of cefpodoxime during freezing and thawing**

| Freeze-thaw times | Spiked concentrations (ng/mL) | | |
| --- | --- | --- | --- |
|  | 50 | 500 | 4000 |
| 0 | 53.1 | 543.15 | 3904.58 |
|  | 51.8 | 560.15 | 3928.3 |
|  | 49.38 | 535.03 | 4063.94 |
| 1 | 54.08 | 549.28 | 4494.7 |
|  | 51.61 | 548.39 | 4019.08 |
|  | 54.76 | 430.32 | 4014.84 |
| 2 | 44.7 | 552.29 | 4212.91 |
|  | 55.04 | 505.58 | 4261.2 |
|  | 53.16 | 571.11 | 4129.07 |
| 3 | 46.37 | 488.69 | 4151.04 |
|  | 56.96 | 471.31 | 3501.72 |
|  | 53.86 | 554.53 | 4355.25 |

**Table S4. Stability of cefpodoxime stored at room temperature for 24h**

| Storage time at room temperature (hour) | Spiked concentrations (ng/mL) | | |
| --- | --- | --- | --- |
|  | 50 | 500 | 4000 |
| 0 | 51.96 | 537.38 | 3462.73 |
|  | 51.79 | 490.03 | 3384.20 |
|  | 51.78 | 484.46 | 3547.33 |
| 24 | 52.00 | 497.00 | 3724.53 |
|  | 53.45 | 539.49 | 3747.88 |
|  | 53.88 | 528.22 | 3719.09 |
